# “Empowerment for Us by Us (E4UBU)”: Developing a Model of Empowerment Using Feminist Participatory Methods with LBQT+ Persons Assigned Female at Birth in Western Kenya

**DOI:** 10.3390/ijerph21070948

**Published:** 2024-07-19

**Authors:** Heather M. Tucker, Rebecca Odhiambo, Laura Jadwin-Cakmak, Anita Mbanda, Ashley Lacombe-Duncan, Caroline Rucah, Ini-Abasi Ubong, Cynthia Akoth Ouko, Wilson Odero, Gary W. Harper

**Affiliations:** 1 Center for Global Health Equity, School of Medicine, University of Michigan, Ann Arbor, MI 48105, USA; 2 Women Empower and Mentor All, CBO, Kisumu 40100, Kenya; 3 School of Public Health, Health Behavior and Health Education, University of Michigan, Ann Arbor, MI 48109, USA; ljadwin@umich.edu (L.J.-C.); iubong@umich.edu (I.-A.U.); gwharper@umich.edu (G.W.H.); 4 School of Social Work, University of Michigan, Ann Arbor, MI 48109, USA; lacombed@umich.edu; 5 Western Kenya LBQT Feminist Forum, Kisumu 40100, Kenya; 6 Homa Bay LBQT Womxn Network, Homa Bay 40300, Kenya; 7 School of Medicine, Maseno University, Kisumu 40100, Kenya

**Keywords:** empowerment, feminist participatory methods, sexual and gender minority persons, Kenya, lesbian, bisexual, queer, transgender men, sexual and gender-based violence, sexual and gender minority-based violence, human rights violations, sexual and reproductive health and rights, mental health

## Abstract

Lesbian, bisexual, queer, trans and other gender diverse persons assigned female at birth (heretofore referred to as “LBQT+ persons”) in Western Kenya experience intersectional oppression and stigma. This stigma can manifest in acts of sexual and gender-based violence (SGBV) and sexual and gender minority (SGM)-based violence, as well as various forms of discrimination—all of which have been linked to disproportionately higher levels of negative health outcomes for this group. Despite these challenges, many LBQT+ persons have been able to gain personal and collective power and thrive in this oppressive environment. The Empowerment for Us by Us (E4UBU) project is a mixed methods feminist participatory research study focused on exploring how LBQT+ persons conceptualize and define empowerment for themselves, and to understand their perspectives on how feelings of power and powerlessness influence their physical and mental health. This paper focuses on data from the first phase of the study, in which qualitative in-depth interviews were conducted with 40 LBQT+ persons (ages 19 to 50) from Kisumu and Homa Bay in Western Kenya. A participatory interpretive phenomenological analysis was conducted to understand the lived experiences of LBQT+ persons as they navigate intersectional oppression and its influence on their experiences of empowerment and subsequent health outcomes. Findings from this analysis were presented to two different focus groups composed of participants who had participated in the in-depth interviews to gather their insights on the interpretations of the interviews as a form of member checking. Findings revealed that “empowerment” was not experienced and viewed by LBQT+ persons as a monolithic construct, but rather a process through which LBQT+ persons are able to transform negative forces of intersectional oppression and powerlessness into experiences of power and subsequent individual and collective action and impact—all leading to improved mental health and well-being. This process is facilitated at several junctures by participatory seeking and attainment of community-appropriate resources at multiple socio-ecological levels that, when accessed with sufficient intensity, frequency, and duration, enhance one’s journey through the process of empowerment. These facilitation junctures are viewed as likely points of focus for public health intervention. Analysis also revealed that the process of empowerment is dependent on the context within which the process is occurring, the specific issues being faced, and the population of focus. Recommendations for how this model can be used for future research and practice to improve the lives of LBQT+ persons in Kenya are discussed.

## 1. Introduction

Homophobia was imported into many Sub-Saharan African (SSA) countries through colonial penal codes which criminalized same sex practices [1]. While some SSA countries have taken steps to remove such penal codes, such as Mozambique and Angola, same sex practices are considered illegal in the majority of countries in SSA, and in some of these countries, such as Mauritania, Sudan, Northern Nigeria, and Southern Somalia, this criminalization includes the death penalty [1]. In the Kenyan context, penal codes inherited through British colonialism criminalize same sex practices, or “un-natural carnal knowledge…against the order of nature” [1]. This criminalization has contributed to a context in which many sexual and gender minority (SGM) persons experience violence, human rights violations (including physical assault, rape and sexual assault), and discrimination in accessing housing, education, health services, and employment [2]. This criminalization is also paired with political, religious, and cultural norms that are largely hetero-normative, and which are further fueled by a more recent politicized homophobia evident in a proposed Family Protection Bill, which would further criminalize SGM persons [3]. This bill is a part of a current trend occurring across a number of former British colonies, through which additional legislation has been proposed by political figures to further criminalize same-sex practices. Research has shown that much of many of these legislative pursuits, including those in Kenya, but also the Ugandan Anti-Homosexuality bill passed in 2023 and a similar bill currently in the Supreme Court in Ghana, have been supported and funded by U.S. Evangelical actors [2]. The harmful effects of the proposed Family Protection Bill on SGM persons in Kenya are already apparent, with increased rates of violence against this population [3]. This social context not only heightens forms of violence against SGM persons but limits their access to health services and amplifies their experiences of discrimination and stigma. Prior research on this population shows that they experience harassment and denial of care from health care workers, and frequently avoid seeking physical and mental health services for fear of discrimination and even violence [4].

LBQT+ persons experience health inequities and human rights violations exacerbated by intersectional oppression related to their gender/gender presentation and sexuality, compared to cisgender (cis) gay and bisexual men in Kenya. Intersectional oppression is the individual experience of structural level privilege and oppression based on social categories (e.g., gender (assigned at birth, expression, identity), sexual orientation, socio-economic status, ethnicity, etc.) [5,6]. In fact, this socio-political landscape can contribute to heightened forms of sexual and gender-based violence, SGM-based violence, family control/coercion, forced marriage, removal of children, a lack of autonomy over one’s sexual and reproductive health choices, discrimination and stigma, and other forms of violence for LBQT+ persons [4,7,8,9,10,11,12,13,14,15]. Studies of LBQT+ persons in Southern Africa, for example, highlight that not only does this group face intersectional stigma and human rights abuses, but are also exposed to heightened levels of forced sexual experiences by men due to their perceived sexual orientation (referred to as “homophobic rape” or “corrective rape”), exposing them to HIV and STIs [16,17]. A research study with SGM individuals in nine African countries (Botswana, Eswatini, Ethiopia, Kenya, Lesotho, Malawi, South Africa, Zambia and Zimbabwe) also shows that LBQT+ persons experienced a heightened frequency of gender-based violence and SGM-based violence compared to the general population [18].

Additionally, LBQT+ persons experience similar forms of oppression as heterosexual cis-gender women in Sub-Saharan Africa (SSA), while facing additional stressors related to their sexuality, which can lead to increased rates of negative health outcomes [8,11,12,14,19]. For example, studies have suggested that LBQT+ persons in Kenya are at an elevated risk for negative sexual health outcomes (e.g., unplanned pregnancy, sexually transmitted infections (STI), HIV, unsafe abortions, sexual trauma) due to a lack of appropriate information, patterns of sexual risk behaviors with men, substance use, low utilization of sexual health services, and anti-SGM policies and stigma [12,14,20]. At the same time, LBQT+ persons often struggle to access knowledge regarding their health and human rights and often lack access to sexual health services [21]. Meanwhile, this population faces intersectional stigma in both HIV policy and programming, as well as heterosexual sexual and reproductive health (SRH) services and interventions, creating extreme barriers to accessing appropriate and adequate health care [9,10,11,12,13,15,18,19,20,21,22,23,24]. Given the oppressive social and cultural environment in Kenya and other parts of SSA, LBQT+ persons have few resources to provide support in the face of this adversity [11,13,14].

### Minority Stress and Empowerment

Mounting evidence in Kenya and elsewhere in SSA has demonstrated that persistent stress stemming from anti-SGM prejudice, stigma, and discrimination places SGM individuals at increased risk for negative physical and mental health outcomes [3,4,5,6,7]. This is in alignment with Meyer’s Minority Stress Model, which has documented that adversity and stress increase the development of both physical and mental health challenges for SGM people in the US [8]. Unfortunately, with the exception of one study in Kenya [4], all of the empirical work on the application of the Minority Stress Model to SGM people and communities in SSA has been conducted with those assigned male sex at birth and is often tied to HIV-related research. This has resulted in too little empirical research focused on SGM-related prejudice for LBQT+ persons and too little guidance for developing support for this population.

The Minority Stress Model is also inclusive of and connected to the concept of empowerment. For example, resilience is embedded within the Minority Stress Model and is often viewed as a dynamic process whereby a person can positively adapt and thrive despite risk exposure, high levels of stress, and other forms of adversity [9]. In the context of the Minority Stress Model, the presence of resilience processes can decrease the negative impact of minority stress on health and lead to more positive adaptation to such stress and subsequent improved health outcomes [10,11]. Resilience shares many elements with the concept of empowerment. Although the concept of empowerment has likely existed for centuries and been manifested in different ways across multiple cultures, as an academic term it appears to have been popularized by the community psychologist, Julian Rappaport in 1981 who defined it as “…a process: the mechanism by which people, organizations, and communities gain mastery over their lives” [12]. Zimmerman’s (1995, 2000) model of empowerment describes empowerment as one’s perceived efficacy and control over the social, economic, and political aspects of one’s life, and it is conceptualized as a multi-level construct occurring at the individual, family, organizational, and community levels. Feelings of powerlessness, or lack of control over one’s destiny, emerge as a broad-based risk factor for negative health outcomes, whereas empowerment has been demonstrated as an important promoter of health [13,14]. The promotion of empowerment has been the basis for health promotion programs for an array of populations, including SGM people.

Empowerment has been popularized as a term, as well as an outcome in global health work. However, this popularization has transformed the term into a buzzword, often losing its weight in global health and development agendas [23,24]. Scholars have argued, in fact, that the original political emphasis and meaning of the term, which was grounded in social justice and critiques of structural powers, has been lost in its popularization [25]. Additionally, empowerment interventions in global health work may rely on a definition of empowerment that is not only depoliticized in its power analysis, but donor driven, losing any real emphasis on social change and sustainable impact for communities. In fact, without an analysis of power differences between global northern donors using and possibly even defining the term, southern recipients may often have to navigate donor’s conceptions and interpretations of the term to produce empowerment as an output in ways that donors can measure and understand. To effect sustainable change and promote health equity for LBQT+ persons in Kenya, it is critical that researchers and interventionists not impose Western conceptualizations of empowerment and health upon them, but instead focus on local conceptualizations of empowerment and culturally grounded and intersectional approaches to promoting health equity. These may include feelings of empowerment but may also be many other related and unrelated psychological and social supports. Therefore, we need a new model of empowerment to inform interventions and programming based on the lived experiences of LBQT+ persons in SSA. Thus, the Empowerment for Us by Us (E4UBU) project was developed, based in feminist participatory research theory and methods, to better understand cultural conceptualizations of empowerment among LBQT+ persons in Western Kenya. This was a collaboration between the University of Michigan School of Public health, the Western Kenyan LBQT Feminist Forum (WKLFF), and the Homa Bay LBQT Womxn Network (HBL). An overarching goal of this parent study is to create data that will assist in developing policies, programs, and services aimed at promoting health equity among LBQT+ persons in Kenya, and elsewhere in SSA, by understanding Kenyan LBQT+ person’s conceptualizations of empowerment and their perspectives on how empowerment and powerlessness influence their physical and mental health. This paper is focused on describing a “Process of Empowerment Model” specifically for LBQT+ persons in Kenya, which emerged inductively from the narratives and lived experiences of LBQT+ persons who shared their stories with us in the E4UBU study. Preliminary analyses from in-depth qualitative data with LBQT+ persons led to the development of a model of “The Process of Empowerment” based on the community’s understanding and lived experience, and in response to LBQT+ community concerns with current conceptualizations and measurements of empowerment in Kenya and elsewhere in SSA which leave LBQT+ persons invisible. This model represents how LBQT+ persons navigate intersectional oppression and its influence on their experiences of empowerment and subsequent health outcomes.

## 2. Materials and Methods

### 2.1. Methods

Before empowerment and resilience-based prevention and health promotion programs for LBQT+ persons in Kenya (and elsewhere in SSA) are developed and implemented, it is critical to first work in collaborative and participatory ways with LBQT+ persons and communities. Therefore, the approaches in this research project are informed first and foremost using a feminist participatory action research (FPAR) methodological design. FPAR is a combination of feminist frameworks and theory and participatory action research methodologies, with the goal of forming community-based engagement, partnership, and creating social change through policy and action [26]. For example, the LBQT+ community raised concerns with current conceptualizations and measurements of empowerment (including, for example, the Kenyan women’s empowerment index [27]) in Kenya and elsewhere in SSA, which often leave LBQT+ persons invisible. Given this, the team sought to develop a conceptual model of the process of empowerment for LBQT+ persons.

FPAR informs a collaborative approach to research design, collection, and analysis with LBQT+ partners in Kenya and includes the end goal of developing an understanding of empowerment that is defined by the community in order to seek emic (localized, “insider”) solutions to health policy for this population [28]. This is in opposition to more dominant etic (universalizing, “outsider”) approaches that observe phenomena without community participation and integration into the research process, often using measures developed outside of the context, based on the researcher’s conceptualization of the phenomena [28]. This emic approach to understanding empowerment is focused on the lived experience of LBQT+ persons, and therefore develops measures that are meaningful, relevant, and impactful. Also, FPAR principles are informed by a “power-with” approach to collaborative research rather than continuing power imbalances or replicating them within projects, with the aim of co-constructing knowledge with partners [26]. One of the core frameworks of this approach includes an intersectional framework through which to analyze power [29]. Intersectionality is a framework and social theory through which to understand the interlocking and intersecting of relations of power, including privilege and oppression, that affect people’s everyday lives [5,30,31,32]. Intersectional methods are included in the design, collection, and analysis of data collection, and inform the definition of power utilized within the research project. The qualitative phase of the research included individual in-depth interviews and focus group discussions with LBQT+ persons in Kisumu and Homa Bay counties, followed by collaborative co-analysis work by the research team to interpret the qualitative data.

### 2.2. Participants

Participants were purposively sampled to represent diverse characteristics of LBQT+ persons, including a range of sexual orientations, gender identities and expressions, and ages. Participants were screened for eligibility, including (1) age between 18 and 65, (2) assigned female sex at birth, (3) self-identify as LBQ, trans, and/or non-binary, (4) resident of Western Kenya (5) speak English, Swahili, and/or Dholuo. Participants of the qualitative interviews included a total of 40 participants from Homa Bay and Kisumu, Kenya. More than half of the participants identified as lesbian, and more than 20% of participants identified as queer, with 17% identifying as bisexual and 4.9% as other. Participants were between the ages of 19 and 50 years. All participants were assigned female at birth, with 35% identifying as cisgender women, 19.5% as non-binary, 12% as transgender men, 2.4% as intersex, and all other participants choosing the other or unknown options for identifying or locating their gender on a spectrum of options.

### 2.3. Interview and Focus Group Guides

The research team collaboratively created a semi-structured qualitative interview guide for the study. Throughout the trauma-informed training of qualitative interviewers, changes were made to the guide to ensure its applicability and appropriateness to LBQT+ persons in Kisumu. The guide provided an outline for reference, while exploring and discussing with participants their conceptualizations and perspectives. The interview guide included questions focused on the study’s main inquiries: the participant’s conceptualizations of power, powerlessness, resilience, resistance, and empowerment in general and among LBQT+ persons; the primary physical and mental health issues faced by LBQT+ persons in Kenya; and the primary physical and mental health service needs of LBQT+ persons in Kenya. The focus groups were conducted after the initial rapid analysis of the interview data and the model of the process of empowerment was developed. The focus group guide included similar questions from the qualitative interviews, as well as questions to elicit feedback on the process of empowerment model.

### 2.4. Procedures

Data collection was carried out by self-identified LBQT+ members of the research team who are active members of WKLFF and HBL. Interviewers were fluent in English, Kiswahili, and Dholuo. Research team members received training in research ethics, boundaries and self-care, trauma-informed care, maintaining privacy, confidentiality and safety, study protocols, emergency procedures, mandated reporting procedures, data management, and qualitative interviewing. They completed mock interviews that were reviewed using an intensive training tool and received feedback [33]. While all research requires protection of participants, it is especially critical to pay attention to the specific risks to disclosure of same-sex behavior in Kenya, due to the current penal codes which criminalize these acts, therefore this study followed all possible pathways to maintaining confidentiality and anonymity of study participants.

Recruitment of LBQT+ persons was led by local LBQT+ identified team members, with WKLFF members taking a primary role. In the first stage of recruitment, information regarding the qualitative interviews was shared with members of the broader LBQT+ community in Western Kenya through social media announcements on Facebook pages and in WhatsApp groups focused on LBQT+ human rights and health promotion. No shared materials included inclusion criteria or other information that might be stigmatizing and did not include the time and location of any data collection activities. Participants were informed of the time and location of the study activities only after they screened as eligible for the study and indicated that they were interested in participating to ensure safety and confidentiality.

Participants were guided through an informed consent process, wherein they were informed that some of the questions asked may cause discomfort or distress, and participants were assured of their right to refuse to answer any questions they did not wish to answer and were assured of their right to leave an interview prior to completion. Interviews were completed in private spaces to minimize the risk of discomfort and, if the participants demonstrated or articulated signs of distress, the research staff were trained to immediately stop the activity taking place. A referral to a mental health or other support services was available onsite or through referrals to local community providers should participants have needed it. An emergency protocol was in place if the participant revealed information that would have required immediate action (e.g., suicidal intent). Dr. Harper, a clinical psychologist on the research team, was available to provide support if such events occurred.

Four Kenya-based research team members carried out qualitative semi-structured interviews in either Kiswahili, Dholuo or English, depending on the needs of the research participant. Following the interview, interviewers debriefed with participants to see how they were feeling after the interview and identify whether the participant exhibited signs of distress, asked if they were in need of any resources/referrals, and provided 1000 KES (7 USD) for their time and effort. Immediately after the interview, interviewers completed a post-interview survey, where they documented incentive disbursement and entered open-ended notes summarizing and reflecting on key information corresponding to each section of the interview guide shared by that participant. No identifying information was collected from participants. Audio files of interviews were stored in an encrypted file on a password protected laptop computer; they were transcribed verbatim by members of the research team, sections completed in Kiswahili or Dholuo were translated into English, and transcripts were de-identified, removing names and any other information that might be identifying.

Following rapid analysis of the qualitative interviews, in September and November of 2022, two focus group discussions were carried out with 10 participants using the focus group guide described above. The focus groups followed the same procedures as the interviews in terms of recruitment, eligibility screening, informed consent, and research incentive amount. They were conducted by the same trained local LBQT+ members of the research team, in English and Kiswahili as needed. The purpose of the focus group discussions was to present the initial version of the model of the process of empowerment to LBQT+ community members to obtain their feedback on the model. Prior to conducting study activities, ethics approval was obtained from the Maseno University Ethics Review Committee (MUERC) and the University of Michigan Institutional Review Board, and a research license was obtained from the National Commission for Science, Technology and Innovation (NACOSTI) in Kenya.

### 2.5. Data Analysis

Immediately after qualitative interviews were completed, all four Kenya-based interviewers and two US-based research team members engaged in a group-based participatory approach to rapid qualitative analysis developed by the senior author in order to develop a model of empowerment. An interpretive phenomenological framework was used, which describes commonalities among a group of people as they experience a particular phenomenon [34]. This is a primarily inductive analytic approach that allows patterns, themes, and categories to emerge from the data. A phenomenological analysis can guide the analysis towards overlap and patterning amongst a group of people as they have a specific social experience, such as LBQT+ person’s experiences of power, empowerment, powerlessness, and physical and mental health challenges. We used the post-interview summaries completed by interviewers as the primary source of data, as translation and transcription of the interviews was not yet complete. We utilized a rapid approach to analysis because the next quantitative phase of the study built upon findings from the qualitative phase, and we wanted to engage LBQT+ community members in member-checking focus group discussions before beginning survey development. All six members of the analysis team read through the post-interview summaries, taking notes to memo their reflections. The team met and the senior author facilitated a discussion amongst the team that began by asking each analyst to share their reflections on each main topic in the interview guide using four questions: (1) What stood out the most to you? (2) What did you hear that confirmed what you already thought? (3) What was surprising? and (4) What was concerning? These reflections led to discussion on how participants conceptualized empowerment as a process they could go through rather than as an end point or state, and we identified and sketched out how different constructs asked about (i.e., power, empowerment, powerlessness, oppression) were connected in participants’ narratives. We then went back to the interview summaries to identify narratives within participants’ stories that fit with different aspects of the model to confirm and/or update aspects of the model. We then conducted two focus group discussions to obtain input from community members on the initial version of the model. Detailed notes were taken during the focus group discussions; these notes were reviewed by the full team and then together the team made iterative updates to the model based on input given during the focus group discussions and later conversations amongst the research team members. Finally, after interviews were transcribed and translated, another member of the research team reviewed transcripts to confirm the analysis and identified representative quotes. The demographic information that participants provided as part of the screening process was analyzed using simple descriptive statistics (calculating means and/or percentages depending on the measure) using Microsoft Excel.

## 3. Results

### 3.1. Empowerment as a Process

When asked to define empowerment in the qualitative interviews, participants’ responses indicated varied conceptions, sharing that the term was related to: “speaking out”, “courage”, “hard work”, “organizations”, “freedom”, “taking control”, “knowledge”, and “belonging”. These responses indicate a variety of conceptions of empowerment, which often require access resources, may come with disempowering consequences, require social and community support, and are connected to larger structural forces, including the legal context. Often, these forms of empowerment were seen as accessible through LBQT+ focused efforts and organizations.

**Figure 1:**
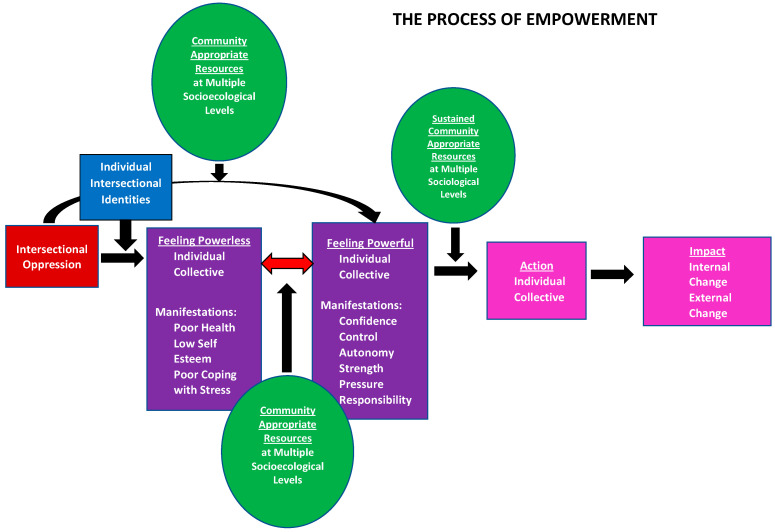
Empowerment as a Process Model.

Overall, we learned from the community that they did not understand empowerment as one central, static construct. Instead, they described a “Process of Empowerment” (Figure 1) driven by access to sufficient, community-appropriate resources across socio-ecological levels that leads to healthy growth and change. Empowerment as a process indicates the layered complicatedness of empowerment for LBQT+ persons; in particular, the need to understand the forms of disempowerment this group experiences, or rather the various barriers to empowerment, considering the intersectional oppression they experience. Through the many different stories and situations shared, participant narratives revealed that while experiences of intersectional oppression could lead to feelings of powerlessness, access to sufficient, community-appropriate resources across socio-ecological levels could allow them to move from feelings of powerlessness to feelings of power. For example, some participants shared experiences of having been sexually assaulted, often when they were young. Those who had not told anyone described feeling powerless and unable to seek help, while other participants who had taken part in groups for LBQT persons and shared their experiences felt as though they had built power. Another participant described how gaining knowledge about LBQT+ communities allowed them to become more vocal about their LBQT+ identity and to build community:

“Yeah, like I’ve gained more knowledge, I’ve become more vocal than I was before. I feel like I feared people, but at least nowadays I can mingle with people. I feel like I know a lot when it comes to like the LGBTQ, unlike, before I just thought that maybe it was just a phase and it’ll go away, but no it ain’t.”

Alternatively, with access to sufficient resources, some LBQT+ persons did not experience feeling powerless despite exposure to intersectional oppression, and instead began from a place of feeling powerful. For example, when asked where power comes from, one participant shared: “I think it’s just within us, me as an LBQ woman. It is inside me, like I have to do this for my own sake for my own sanity”. The feelings of powerlessness caused by intersectional oppression were experienced both on an individual level and on a collective level (e.g., as the local LBQT+ community). Manifestations of powerlessness described by participants included poor health, low self-esteem, and poor coping with stress. Just as feelings of powerlessness could occur at an individual or collective level, so could feeling powerful. Manifestations of feeling powerful included confidence, control, autonomy, strength, pressure, and responsibility.

From a place of feeling powerful, sustained access to resources can lead to taking individual and collective action, which can then lead to impact. Participants described impact as encompassing both internal growth (e.g., perceiving one’s sexuality as valid and a source of pride rather than sinful) or external change (e.g., starting an LBQT+ organization or changing a health system’s policies). For example, one participant described how attending LBQT+ forums became a source of internal growth:

“So with, with time, with the many times I got to attend these meetings, I came to identify myself and know like, no, it’s not a sin, I’m just like other people. So as I continued to attend the forums to date, I feel I’m empowered.”

Movement through this process may stop at any point, and over time, due to internal or external forces. For example, those who attend community forums and connect with other LBQT+ persons (i.e., an accessed resource) may feel powerful as a result. These feelings of powerfulness, which could manifest as increased confidence, may be the “end point” of the process for some attendees, while the process may continue for others who then take individual or collective action, for example, introducing their partner to a family member or working with other LBQT+ community members to mobilize resources to support inclusive mental health services. Additionally, over time, one may move back and forth between feeling powerful and feeling powerless. For example, a local LBQT+ community may collectively feel powerful when engaging in meaningful national advocacy to repeal anti-LGBTQ+ policies, then move to a place of feeling powerless when such policies are not repealed, then again move to a place of feeling powerful when collective efforts to heal and re-start community organizing begin again.

### 3.2. A Process Dependent on Population, Context, and Issue

The process of empowerment is a process dependent on population, context, and issue. Within the LBQT+ community, intersectional identities that determine one’s relationship with power include ethnicity, sexuality, gender expression/identity, marital/partner status, family factors, such as birth order, sexual/reproductive health status, employment status, dependence on or living with biological parents/family. Additionally, within participants’ narratives, we identified how experiences of intersectional oppression, led to feelings of powerlessness. Intersectional oppression experienced by LBQT+ persons included sexuality- and gender-related stressors, as well as general stressors. Sexuality- and gender-related stressors included sexuality, sexual expression, societal sexual identity norms, gender, gender expression, societal gender identity norms; ethnicity, race, and societal cultural norms; family and romantic partnerships; religion and religious institutions; and the government and legal system. General stressors included those related to capitalism and the economy; colonization and minimization of indigenous ways of being; and the physical environment and climate change. What makes this process particularly relevant to the experiences of LBQT+ persons is that these qualitative data show us that sexuality- and gender-related stressors are perpetrated by heterosexual and gendered social norms, the way in which one expresses sexuality or gender, the way in which one identifies one’s sexuality or gender, as well as ethnicity, race and cultural societal norms, family and romantic partnerships, religion and religious institutions, and the government and legal system. These individual intersecting experiences and stressors lead to experiencing intersectional oppression, which can then determine if one is feeling powerless on an individual and or collective level. Therefore, the intersectional identities and types of intersectional oppression that must be considered in a process of empowerment is highly dependent on the population of focus.

How a person or group experiences the process of empowerment is also highly dependent on context and will differ by issue. Issues of focus that were frequently discussed in these interviews included health (mental health, sexual and reproductive health, and broader physical health), economic wellbeing, and religious engagement. It is possible to simultaneously feel powerful and powerless regarding different issues. To take an example related to advocacy, one may have confidence to advocate for sexual and reproductive health services that are inclusive of LBQT+ persons while feeling unable to advocate for LBQT+ inclusivity in educational system policies. Just as one may feel simultaneously powerful and powerless regarding different issues as described above, one may also feel simultaneously powerful and powerless regarding the same issue within different contexts, such as feeling able to advocate for oneself as an LBQT+ person in the workplace in an urban area but unable to advocate for oneself as an LBQT+ person among family in a rural home.

### 3.3. A Process Requiring Sustained, Sufficient Resources

Movement within the process of empowerment is fueled by access to sustained, sufficient resources across socio-ecological levels. Therefore, within this conceptualization of empowerment, resources represent points of potential intervention. The frequency, duration, and intensity of the resources available had an impact on the process of empowerment, as did whether the resources were appropriate to the local culture and community, and the degree to which the resources were participatory in nature, as opposed to being “given” without agency or participation of those receiving the resources. In short, participants’ narratives revealed that not all resources are equal, further emphasizing the importance of context. Local members of the research team emphasized the importance of using appropriate and sustained resources to empower those within the community to do the work, rather than resourcing those from other global or even nearby local communities.

The types of resources identified as important to the process of empowerment included those provided on individual, interpersonal, community, and systems and policy levels. The types of resources described as influencing the process of empowerment were described as necessary on different socio-economic dimensions, including individual, interpersonal, community, systems and policies. Individual resources included money, knowledge, coping skills, attitudes, self-acceptance, and connecting to younger self. Interpersonal resources included money, social support, acceptance by others, who you know, and networking. Community resources included money, community organizing, and visibility. Systems and policy resources included money, social policies, employment, and indigenous knowledge.

Throughout the research team’s iterative discussions to develop the “Process of Empowerment Model”, we identified—both from the data and from the Kenyan team members’ personal experiences with advocacy and public health work—four important questions to ask when developing or implementing a resource-focused intervention: (1) who is providing resources? (2) who determines what is needed? (3) how are the resources provided? (4) and how are the resources accounted for? These questions should be posed to interrogate the balance of power within an intervention, and the answers to these questions should elucidate whether (or the degree to which) resources provided are community-appropriate, participatory, and sufficient.

## 4. Discussion

The Empowerment for Us by Us (E4UBU) project was developed to better understand cultural conceptualizations of empowerment among LBQT+ persons in Western Kenya, based on feminist participatory methods. The model highlights how LBQT+ persons are particularly vulnerable to disempowering effects of intersectional oppression. The preliminary research analysis above also indicates that empowering resources, defined by LBQT+ persons, are critical in order to enable the process of empowerment, considering the current context in Kenya.

As the Process of Empowerment model shows, the individual qualitative interviews indicated that empowerment is highly contextual, but also local/cultural. The semi-structured interviews explored LBQT+ persons’ conceptualizations and definitions of empowerment to understand their perspectives on how empowerment and powerlessness influence their physical and mental health. These critical perspectives highlight the importance of defining empowerment by the community, and based on community needs and desires, rather than from donors’ desires about what communities want, which can often take place in development and global health interventions.

Because empowerment is often used as a universalized buzzword in global health and may be highly influenced by donor or institutional agendas, the definitions and conceptions of empowerment, and its meaning to communities is critical. This emic understanding of the concept is not only important to determine how access to power works, but also what resources and changes are relevant for people to both access power that they already may possess and to challenge power that is oppressive. As shown in the E4UBU empowerment model, access to resources over time in a way that is sustainable is central for this definition of empowerment to become a reality. Not only does the model offer a localized, contextualized, and nuanced conception of empowerment that is specific to Western Kenyan LBQT+ persons experiencing intersectional oppression, but it also offers insight into the notion that empowerment is a process and an outcome, and access to resources and social change mechanisms are often the route to change for communities experiencing multiple structural forms of oppression.

Those programs, policies, and interventions aimed towards providing better health outcomes and well-being for LBQT+ persons in SSA can use this process model as a point of reference to create relevant and sustainable solutions for interventions in the context. Interventions that are guided by this model and the specific lived experiences of this group can be informed by specific information on the barriers and successes available to this group in the SSA context, and the specific resources needed over time to affect impact for this group. One specific example of a program that has grown from this process is the WEMA Womxn project, a manualized peer-based mental health intervention designed by and for LBQT+ persons in Western Kenya (Tucker et al. forthcoming). WEMA Womxn is aimed at increasing the mental health and well-being of LBQT+ persons in Western Kenya, to increase resilience in the face of the various forms of intersectional stigma and oppression faced by this group. The intervention is a trauma-informed six session intervention that is grounded in Minority Stress Theory and Critical Consciousness and utilizes cognitive behavioral activities that help participants to explore and address mental health, stress, coping with stress, coping with LGBTIQ+ minority oppression and stigma, fostering healthy social connections, and building community and social capital. The manual was developed so that local members of the LGBTIQ+ community can deliver the intervention with limited training and supervision, therefore sustaining the intervention as a resource available over time.

## 5. Conclusions

The current social and cultural environment in Kenya and other parts of SSA has been documented as extremely oppressive for LBQT+ persons, who have few resources to provide support in the face of this adversity. In addition, there is very little research focused on LBQT+ persons and their experiences of oppression, and very little guidance for developing support for this population. However, LBQT+ persons have community-based solutions to the intersectional oppression they are facing. Grounded in feminist participatory methodologies with LBQT+ persons from the outset, this preliminary analysis from the qualitative phase of the E4UBU study illuminated that empowerment is a complicated, ongoing, dynamic process, driven by access to resources over time at different socio-ecological levels. The E4UBU data also highlights that, despite experiences of intersectional oppression, LBQT+ persons can access power at multiple socio-ecological levels, which can lead to changes for both individuals and communities. However, while empowerment as a process is dependent on population, context, and issue, it is highly reliant on sustained, appropriate, sufficient resources, and can stop at any time depending on access to such resources. With these lessons learned, the E4UBU study can ideally be used as a road map to inform research, policy, and interventions, which can then enable sustainable change and health equity that is grounded in lived experience.

## Data Availability

The data presented in this study may be available on request from the corresponding author. The data are not publicly available due to the sensitive and potentially identifying nature of the qualitative data collected.
